# Supplementary material for: miR-335 Restrains the Aggressive Phenotypes of Ovarian Cancer Cells by Inhibiting COL11A1
Source: Cancers (Basel). 2021 Dec 13;13(24):6257. doi: 10.3390/cancers13246257 (PMC8699536; doi:10.3390/cancers13246257)
Supplement: Supplementary file 1 [file cancers-13-06257-s001.zip › cancers-1478321-supplementary.pdf]

miR-335 restrains the aggressive phenotypes of ovarian cancer cells by inhibiting COL11A1

Yi-Hui Wu [1,2], Yu-Fang Huang [3], Tzu-Hao Chang [4], Pei-Ying Wu [5], Tsung-Ying Hsieh [6], Sheng-Yen Hsiao [7] and Soon-Cen Huang [8#] and Cheng-Yang Chou [9#*]

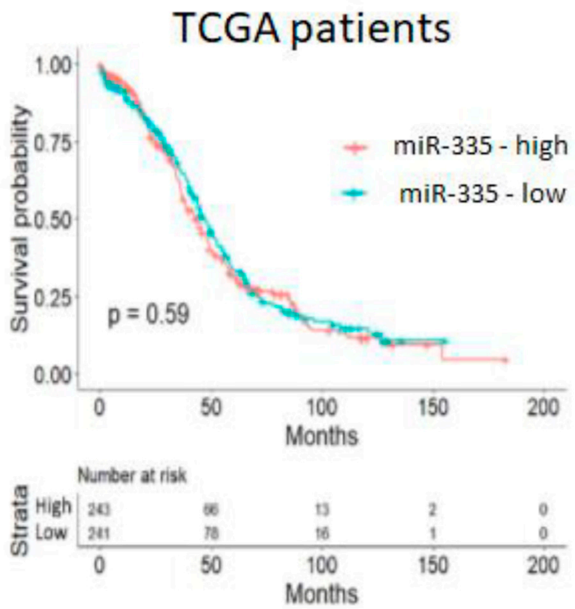

Figure S1. Long-termoverall survivalsinthe TCGApatients (n= 483). Kaplan–Meier curves stratified by the miR-335mRNA level and assessed by log-rank testare illustrated.

Table S1. Complementary oligonucleotides used to construct double-stranded, mutant miR-335-binding sites of COL11A1 3`-UTR or PDK1 3`-UTR.

	Sequence
COL11A1 region 1 (Mut) F	GTAATTATTGGGTGTACAGTTCTATACT
COL11A1 region 1 (Mut) R	AGTATAGAACTGTACACCCAATAATTAC
COL11A1 region 2 (Mut) F	AAAATATTATGCAGGGTGATGCTTTATT
COL11A1 region 2 (Mut) R	AATAAAGCATCACCCTGCATAATATTTT
COL11A1 region 3 (Mut) F	GTTAGATTGGCTTATCAATCAAGTTCTT
COL11A1 region 3 (Mut) R	AAGAACTTGATTGATAAGCCAATCTAAC
PDK1 (Mut) F	TGGCTTGGTCATTGTAGGGCGTGGTTGT
PDK1 (Mut) R	ACAACCACGCCCTACAATGACCAAGCCA

Table S2. MicroRNA target interactions of COL11A1 gene.

COL11A1	microRNA target interactions	miRNAs

**3'UTR**	596	162
**5'UTR**	1238	293
**CDS**	8874	1744

Table S3. Top 10 negative correlated microRNAs.

TCGA_OV_Top10_neg_Cor	Spearman
hsa-miR-508-3p	-0.557299073
hsa-miR-509-3p	-0.538068643
hsa-miR-508-5p	-0.529931704
hsa-miR-125b-1-3p	-0.510822844
hsa-miR-106b-5p	-0.510332904
hsa-miR-410-3p	-0.494012796
hsa-miR-409-3p	-0.491745859
hsa-miR-1228-5p	-0.449826617
hsa-miR-101-3p	-0.446658052
hsa-miR-616-3p	-0.425184671

Table S4. MicroRNAs with negative correlation and predicted microRNA target interactions.26 .

miRNAs	MTIs	Correlation with COL11A1 (Spearman)
hsa-miR-509-3p	4	-0.538068643
hsa-miR-30e-5p	2	-0.224647756
hsa-miR-877-5p	8	-0.204469492

Table S5. Correlations between miR-335 and COL11A1 mRNA 28 levels.

		N	miR-335 mRNA level	*P*
Non-cancer		23	2306.63 ± 9604.63	0.002
Cancerous		137	182.39 ± 1915.05	
COL11A1	low	63	856.06 ± 4289.9	<0.001
	high	74	3.90 ± 279.43	

Data was presented as median ±interquartile range(IQR); Data was analyzed by Mann-Whitney U test; COL11A1 level <1446.67 (low) and ≥1446.67(high).

Table S6. TCGA data.

Characteristic	Number of patient
Stage	
I	1
II	27
III	373

IV	80
NA	4
Age	
≧54	326
<54	157
Disease status	
Alive	217
Death	268
Debulking surgery	
Optimal surgery	313
Suboptimal surgery	115

Table S7. TCGA miRNA data.

	Mean	Early stage	Advanced stage	P adjust	Significant
**TCGA miR-335**	197.005268	142.29 [66.91-196.38]	106.62 [56.16-141.23]	0.000336	Downregulated in advance stage

Table S8. TCGA miRNA data.

	Mean	Advanced stage Optimal debulking surgery	Advanced stage suboptimal debulking surgery	P adjust	Significant
**TCGA miR-335**	153.6643017	150.74 [52.06-158.26]	161.25 [69.68-155.25]	0.5150	No significance

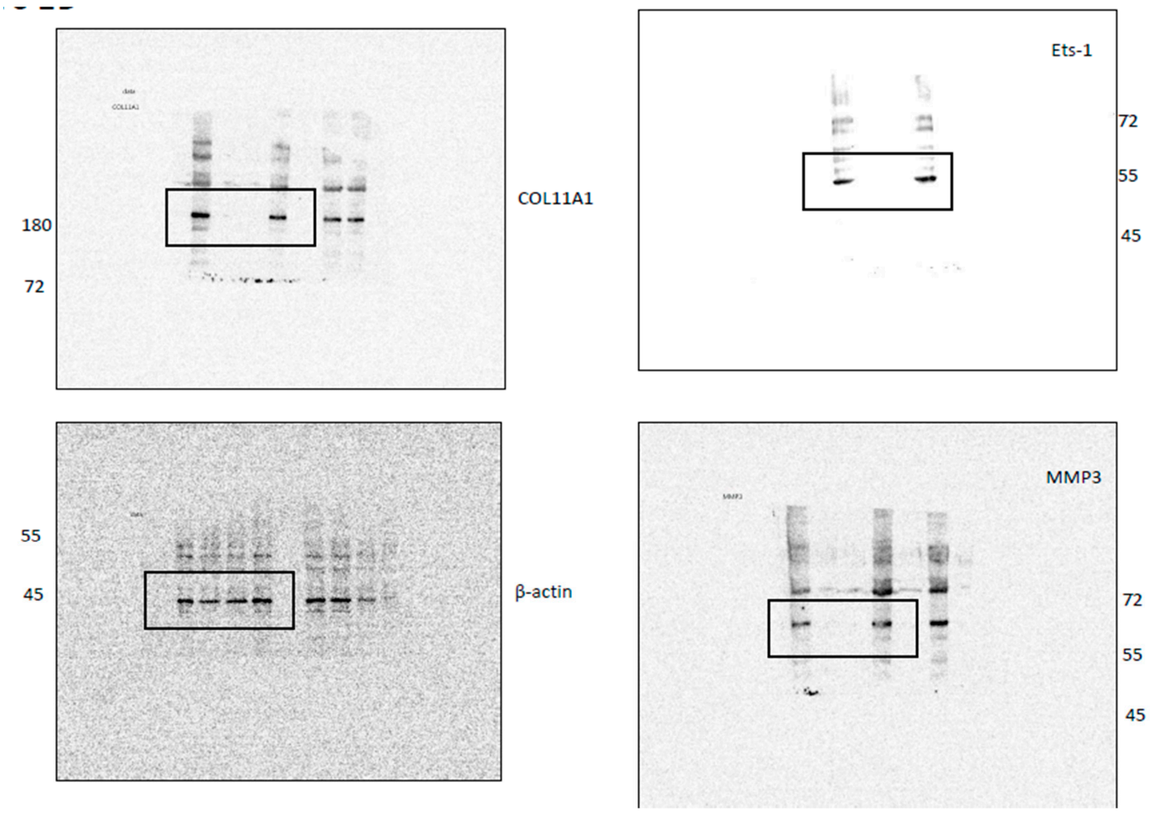
COL11A1
180
72
Ets-1
72
55
45
55
45
β-actin
MMP3
72
55
45
(**A**)

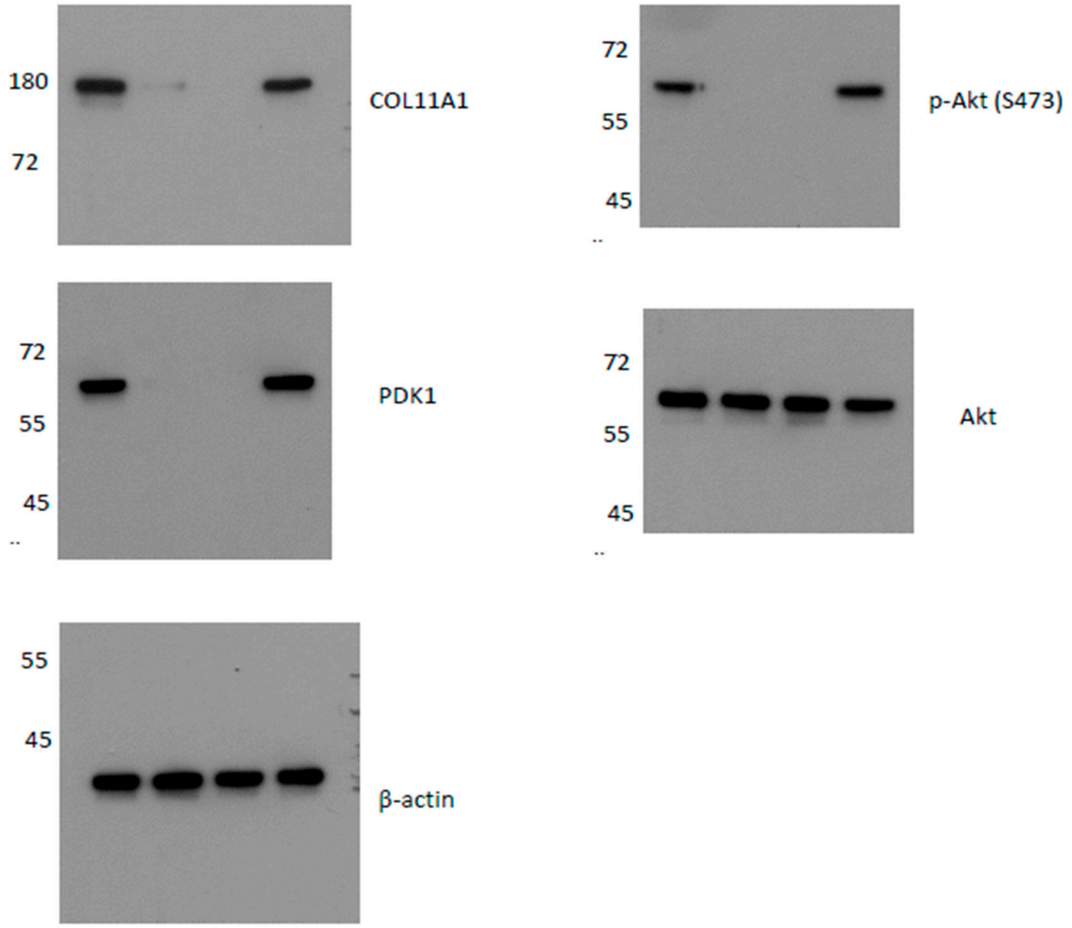
180
72
COL11A1
72
55
45
p-Akt (S473)
72
55
45
PDK1
72
55
45
Akt
55
45
β-actin
(**B**)

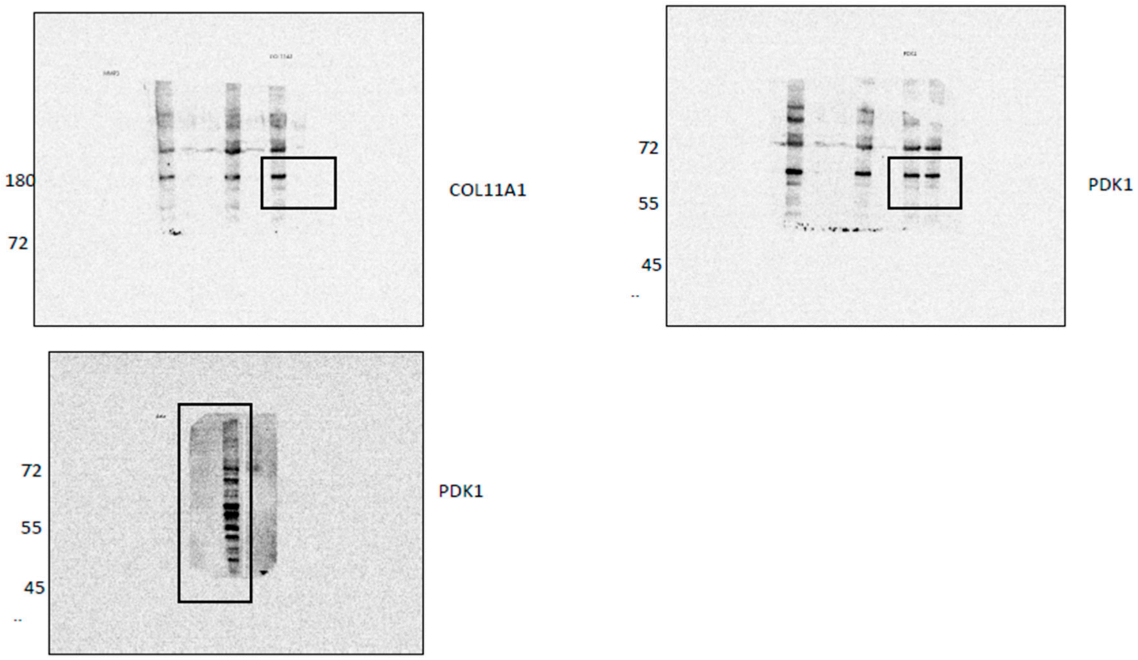

(**C**)

Figure S2. Western blot (**A**): reference to Figure 2E; (**B**): reference to Figure 3B; (**C**): reference to Figure 3D.
